# Medical students’ attitude towards influenza vaccination

**DOI:** 10.1186/s12879-015-0929-5

**Published:** 2015-04-15

**Authors:** Birthe A Lehmann, Robert AC Ruiter, Sabine Wicker, Gretchen Chapman, Gerjo Kok

**Affiliations:** Department of Work & Social Psychology, Faculty of Psychology and Neuroscience, Maastricht University, PO Box 616, 6200 MD Maastricht, The Netherlands; Betriebsärztlicher Dienst, Klinikum der Johann Wolfgang Goethe-Universität Frankfurt, Theodor-Stern-Kai 7, 60590 Frankfurt, Germany; Department of Psychology, Rutgers University, 152 Frelinghuysen Road, Piscataway, NJ 08854-8020 USA

**Keywords:** Medical students, Hospital, Influenza vaccination, Social-cognitive predictors

## Abstract

**Background:**

Influenza vaccination is recommended for all healthcare personnel (HCP) and most institutions offer vaccination for free and on site. However, medical students do not always have such easy access, and the predictors that might guide the motivation of medical students to get vaccinated are largely unknown.

**Methods:**

We conducted a cross-sectional survey study among pre-clinical medical students in a German University hospital to assess the social cognitive predictors of influenza vaccination, as well as reasons for refusal and acceptance of the vaccine.

**Results:**

Findings show that pre-clinical medical students have comparable knowledge gaps and negative attitudes towards influenza vaccination that have previously been reported among HCP. Lower injunctive norms and higher feelings of autonomy contribute to no intention to get vaccinated against influenza, while a positive instrumental attitude and higher feelings of autonomy contribute to a high intention to get vaccinated. The variables in the regression model explained 20% of the variance in intention to get vaccinated.

**Conclusions:**

The identified factors should be addressed early in medical education, and hospitals might benefit from a more inclusive vaccination program and accessibility of free vaccines for their medical students.

## Background

Annual influenza epidemics are a major public health problem causing severe morbidity and mortality, especially in high risk groups. High risk groups include children younger than 2, people over the age of 65, and patients with medical conditions that make them more likely to suffer from influenza-related complications [1,2]. Healthcare personnel (HCP) can serve as vectors in the transmission of influenza to vulnerable patients and are therefore recommended to get vaccinated against influenza annually [3-6]. Most hospitals and medical institutions offer their HCP vaccination for free and on site. In contrast, such easy access to the vaccine is not always offered to their medical students [7], even though they have regular patient contact throughout their education.

Research has repeatedly shown that a large proportion of HCP have unfavourable attitudes towards influenza vaccination, and the reasons for rejecting or accepting influenza vaccination have been examined extensively [7-16]. However, only a few studies included medical students [7,8,16]. Finding out about the factors that predict medical students’ motivation to get vaccinated against influenza can have important implications for the education of these students in terms of developing a favourable attitude towards influenza vaccination and addressing knowledge gaps. Moreover, it can have implications for hospitals in terms of motivating their students to get vaccinated against influenza annually and to include them in vaccination programs. After all, medical students have patient contact throughout their education, and they are the physicians of the future. Therefore, we conducted a cross-sectional survey to investigate the social cognitive variables that predict the influenza vaccination intention of medical students.

The few existing studies that included medical students [7,8,16] exclusively investigated post-hoc reasons for acceptance and refusal of the vaccine, while the predictors that might guide the motivation of medical students are largely unknown. In the current study, we therefore utilized measures of constructs from the Reasoned Action Approach (RAA) in our cross-sectional survey [17]. The RAA is a social cognition model of human behaviour, which proposes that the motivation or intention to perform a behaviour is caused by attitudes, perceived norms, and perceived behavioural control. Attitudes are a person’s overall evaluation of the anticipated advantages and disadvantages resulting from performing a behaviour. Perceived norms refer to the anticipated approval or disapproval of significant others concerning performance of a health behaviour and also whether comparable others perform the behaviour themselves. Finally, perceived behavioural control refers to the belief regarding degree of perceived capacity and autonomy in performing the behaviour. The RAA has been successfully used to predict the influenza vaccination intentions and behaviour of HCP [18]. Attitude has been shown to be one of the strongest predictors of the intention to get vaccinated. Medical students who are lower in hospital hierarchies might be more susceptible to perceived norms than other HCP groups.

This study investigated the social cognitive variables that predict medical students’ intention, as well as reasons for refusal and acceptance to get vaccinated against influenza. Results of this cross-sectional study can assist in the development of future educational programs for medical students and can provide advice to hospitals about how to include medical students into their annual HCP vaccination programs.

## Methods

### Participants and procedure

Pre-clinical medical students at the University Hospital Frankfurt have to attend an occupational health screening before their preliminary medical examination, at the end of their second year. In May 2012 and 2013, these students were asked to fill in a cross-sectional survey about the factors influencing the decision to get vaccinated against influenza (N = 264 in 2012, N = 279 in 2013).

### The questionnaire

The first questionnaire conducted in 2012 consisted of 18 questions, answered on 7-point Likert scales, unless otherwise indicated. Demographic measures included age, sex and current vaccination status. Table 1 provides an overview of the questions about social cognitive variables utilized in the survey. Additionally, all participants were asked to indicate which of a set of 8 *facilitating factors* of influenza vaccination would apply to them (self-protection, patient protection, protection of family and friends, work ethic to not infect anyone, advice from a medical expert, to set a positive example for patients, vaccination available for free, vaccination is safe) and exclusively non-immunizers were asked to indicate which of a set of 9 *inhibiting factors* apply to their decision to not get vaccinated (no specific risk, influenza is not a serious disease, fear of side-effects, vaccination provides insufficient protection, influenza vaccination was never offered to me, vaccination could cause flu, no possibility to get vaccinated, medical contraindication, fear of needles). Multiple answers were possible.

**Table 1:** **Overview of constructs measured by the survey**

**Variable**	**Questions**
Instrumental attitude	Getting vaccinated against influenza every year in October/ November would be: very good – very bad
Experiential attitude	When I think of getting vaccinated against influenza annually, it makes me: very anxious – not at all anxious
Injunctive norm	Most people who are important to me think I should get vaccinated against influenza annually. agree – disagree
Descriptive norm	Most physicians get vaccinated against influenza annually. very unlikely – very likely
Capacity	I am confident that I can get vaccinated against influenza next October/ November, if I want to. true – false.
Autonomy	Getting vaccinated against influenza annually is up to me. agree – disagree
Behavioral beliefs (self-protection and patient protection)	Getting vaccinated against influenza annually will result in fewer influenza infections and less work absenteeism. very likely – very unlikely; Getting vaccinated against influenza annually will prevent at-risk patients from getting influenza. true – false
Knowledge about recommendations	I know about the national recommendations for health care workers to get vaccinated against influenza annually, in order to protect themselves and patients against influenza infections. true – false
Injunctive normative belief	My future employer will think that: I should – I should not get vaccinated against influenza annually.
Control belief	I expect that most hospitals enable their employees to get vaccinated against influenza annually at work. very likely – very unlikely.
Intention	I intend to get vaccinated against influenza next October/ November. very unlikely – very likely.
Barrier (high workload)	Imagine that on the day you get offered influenza vaccination at work, you are under a lot of time pressure and barely have time to take a break. How likely is it that you will still get vaccinated against influenza? very likely – very unlikely

Some of the questions used for analyses came from an attached questionnaire utilized by one of the co-authors of this study. Due to an unforeseen change in the set-up of the study, these questions were missing for the 2013 sample. The questionnaire conducted in 2013 was missing the demographic questions and the questions about facilitating and inhibiting factors of influenza vaccination, resulting in 14 questions from the previous questionnaire.

### Data analysis

SPSS 20.0 was used to analyse the data. Following a descriptive analysis of the sample (frequencies), univariate associations between intention and social cognitive variables were analysed with Pearson correlation coefficients. Significance was set at *p* ≤ .05. Intention was not normally distributed, and thus we classified responses into three groups; no intention to get vaccinated against influenza (0 = 1.0-2.0), not having made a clear decision about vaccination (1 = 2.5-5.5), and a high intention to get vaccinated (2 = 6.0-7.0). Therefore, multinominal logistic regression was used to examine the effects of the independent variables on the probability of (1) having no intention to get vaccinated vs. not having made a clear decision and (2) having a high intention to get vaccinated vs. not having made a clear decision. We checked for multicollinearity by inspecting the VIF and Tolerance values.

## Results

### Descriptive statistics

The 2012 sample consisted of 264 pre-clinical medical students (see Table 2), with 91 males (34.5%) and 173 females (65.5%) and a mean age of 23 years (range 20 to 47). Of these students, 34 (12.9%) were vaccinated against influenza. The 2013 sample consisted of 279 German medical students. In 2012, 107 (41%) of the participants reported having no intention to get vaccinated against influenza, 99 (37%) indicated not having made a clear decision, and 57 (22%) reported a high intention to get vaccinated. In 2013, these numbers were highly similar with 115 (41%), 100 (36%), and 63 (23%) respectively. Table 3 shows the facilitating and inhibiting factors that participants of the 2012 sample were asked to choose from as reasons to accept or reject influenza vaccination. Among the facilitating factors, self-protection, patient protection, and the protection of family and friends were the most frequently chosen reasons in favour of influenza vaccination by both non-immunizers and immunizers. Logistic regression analysis revealed that the facilitating factors account for 25% of the explained variance in vaccination uptake (Nagelkerke R^2^ = .25). Among the inhibiting factors, not being at a specific risk was chosen most often by non-immunizers (N = 116, 50.4%), followed by thinking that influenza is not a serious disease (N = 51, 21.7%). Other relatively common reasons were fear of side-effects (N = 46, 20%), thinking that vaccination provides insufficient protection (N = 44, 19.1%), and that influenza vaccination had never been offered (N = 39, 17%). Less common reasons were the belief that the vaccination could cause flu (N = 13, 5.7%), not having had the possibility to get the vaccination (N = 6, 2.6%), a medical contraindication (N = 5, 2.2%), and fear of needles (N = 2, 0.9%).

**Table 2:** **Students’ demographics and vaccination characteristics (2012 and 2013)**

	**2012 (N = 264, 48.6%)**	**2013 (N = 279, 51.4%)**	**Total (N = 543)**
Male	91 (34.5)	N.A.	N.A.
Female	173 (65.5)	N.A.	N.A.
Mean age (S.D.)	23.1 (3.32)	N.A.	N.A.
Vaccinated	34 (12.9)	N.A.	N.A.
No intention	107 (41)	115 (41)	222 (41)
No clear decision	99 (37)	100 (36)	199 (36.7)
High intention	57 (22)	63 (23)	120 (22.1)

Data are reported as number of participants (%).

Data not available (N.A.).

**Table 3:** **Facilitators and inhibitors of influenza vaccination (2012 sample)**

**Factors**	**Not vaccinated (N = 230, 87%)**	**Vaccinated (N = 34, 13%)**	**Total (N = 264)**	**OR (95% CI)**	**P-value**
*Facilitators*					
Self-protection	154 (67)	32 (94.1)	186 (70.5)	9.62 (2.12-43.55)	.003
Patient protection	140 (60.9)	30 (88.2)	170 (64.4)	6.08 (1.83-20.13)	.003
Family and friends	106 (46.1)	12 (35.3)	118 (44.7)	0.18 (0.07-0.46)	<.001
Work ethics	65 (28.3)	14 (41.2)	79 (29.9)	1.53 (0.59-3.94)	.38
Medical advice	42 (18.3)	4 (11.8)	46 (17.4)	0.57 (0.16-2.09)	.40
Set positive example	36 (15.7)	6 (17.6)	42 (15.9)	0.87 (0.25-2.95)	.82
Free of charge	34 (14.8)	4 (11.8)	38 (14.4)	0.72 (0.19-2.73)	.63
Flu shot is safe	32 (13.9)	7 (20.6)	39 (14.8)	2.78 (0.79-9.75)	.11
*Inhibitors*					
No specific risk	116 (50.4)	-	116 (43.9)		
No serious disease	51 (21.7)	-	51 (19.3)		
Fear of side-effects	46 (20)	-	46 (17.4)		
Insufficient protection	44 (19.1)	-	44 (16.7)		
Never offered	39 (17)	-	39 (14.8)		
Causes flu	13 (5.7)	-	13 (4.9)		
No possibility	6 (2.6)	-	6 (2.3)		
Medical contraindication	5 (2.2)	-	5 (1.9)		
Fear of needles	2 (0.9)	-	2 (0.8)		

Data are reported as number of participants (%).

Note R^2^ = .25.

### Correlates of intention to get vaccinated

Table 4 shows the correlates of intention to get vaccinated of students in 2012 in the bottom half and students in 2013 in the upper half. A small effect is *r* = .10-.23, a moderate effect *r* = .24-.36 and a large effect is *r* ≥ .37 [19]. In 2012, we found a moderate positive univariate association with intention for instrumental attitude, and small positive associations with injunctive norm and high workload. In 2013, strong positive associations were found for instrumental attitude and injunctive norm. A moderate negative association was found for the barrier of high workload. Experiential attitudes, descriptive norm, self-protection, patient protection, knowledge about national recommendations, injunctive belief, and control belief all showed a small univariate association with intention in 2013 (see Table 4).

**Table 4:** **Correlations of intention with social cognitive factors in 2012 and 2013**

	**1**	**2**	**3**	**4**	**5**	**6**	**7**	**8**	**9**	**10**	**11**	**12**	**13**
1. Intention	*1*	.39**	.23**	.39**	.20**	*.12*	*-.03*	.17**	.12*	.14*	.13*	.22**	-.34**
2. Instrumental attitude	.26**	*1*	.31**	.56**	.27**	.27**	*.05*	.38**	.30**	.27**	.27**	.39**	-.51**
3. Experiential attitude	*.12*	.31**	*1*	.20**	.19**	*.10*	*.10*	.17**	.13*	.15*	*.06*	*.11*	-.25**
4. Injunctive norm	.21**	.49**	.18**	*1*	.17**	.14*	*.05*	.36**	.18**	.16**	.22**	.28**	-.36**
5. Descriptive norm	*.06*	.26**	.16**	.36**	*1*	.17**	*.01*	.16**	.12*	.18**	.24**	.17**	*-.11*
6. Capacity	*.01*	.24**	.36**	.19**	.29**	*1*	.25**	.24**	*.08*	*.08*	.13*	.16**	*-.17*
7. Autonomy	*-.10*	*-.03*	*.11*	*-.06*	*.06*	.14*	*1*	*.07*	*.09*	*.11*	*-.00*	*.05*	*.00*
8. Self-protection	*.08*	.37**	*.11*	.53**	.23**	.23**	*.07*	*1*	.24**	*.06*	.12*	.14*	-.26**
9. Patient protection	*-.01*	.35**	.16*	.41**	.26**	.23**	*.10*	.60**	*1*	.22**	.31**	.23**	-.22**
10. Recommendation	*.11*	.32**	.18**	.31**	.19**	.27**	*-.02*	.38**	.20**	*1*	.24**	.20**	*-.12*
11. Injunctive belief	*.09*	.30**	.22**	.30**	.28**	.33**	*.07*	.33**	.46**	.34**	*1*	.45**	-.23**
12. Control belief	*.04*	.29**	.26**	.16**	.28**	.30**	*.06*	.35**	.43**	.27**	.52**	*1*	-.33**
13. High workload	.13*	.39**	.21**	.23**	.15*	.27**	-.18**	.30**	.31**	.30**	.28**	.28**	*1*

Note: N = 263 for bottom half (2012 sample) and N = 278 for upper half (2013 sample).

*p < .05, two-tailed; **p < .001, two-tailed.

### Multinominal logistic regression

In the influenza season 2012/13, some influenza vaccines of the provider Novartis had to be retracted temporarily in some European countries, including Germany. Flocculation had been observed in some vaccines, which is the formation of visible clusters due to clumps of protein particles, and it had been recalled by the provider as a safety measure. This could have potentially affected the comparability of student responses in 2013 and we formally tested for differences between both samples because of this incident by examining possible interaction effects of the predictor variables with sample (2012 vs. 2013). Except for two interaction terms (high workload for no intention vs. no clear decision; descriptive norm for high intention vs. no clear decision), no significant interaction terms were found, suggesting no systematic evidence for an impact of sample (i.e. Novartis incident) on the relationships of the predictor variables with intention. The interaction terms were therefore removed from the model after which the analyses were repeated.

Results of the multinominal logistic regression across samples are shown in Table 5. Medical students with lower injunctive norms and higher feelings of autonomy were significantly more likely to have no intention to get vaccinated vs. not having made a clear decision. Having a positive instrumental attitude and higher feelings of autonomy significantly increased the probability of having a high intention to get vaccinated vs. not having made a clear decision. The variables in the regression model explained 20% of the variance in intention (Nagelkerke Pseudo R^2^ = .20), with a classification accuracy of 55.2%. There was no significant contribution of sample to the prediction of intention, indicating that there was no difference in intention between both samples after the Novartis incident took place. Inspection of the VIF and tolerance values did not indicate any cause for concern with regard to multicollinearity.

**Table 5:** **Multinominal logistic regression**

**Predictors**	**r**	**b**	**S.E.**	**Wald**	**P**
*No intention vs. no clear decision (N = 421)*					
Instrumental attitude	-.23**	−1.47	.09	2.85	.09
Experiential attitude	-.10*	-.10	.07	2.01	.16
Injunctive norm	-.28**	-.24	.07	11.23	.001
Descriptive norm	-.04	.06	.08	.62	.43
Capacity	.01	.03	.05	.33	.57
Autonomy	.20**	.33	.09	13.30	<.001
Self-protection	-.10*	.01	.07	.03	.87
Patient protection	-.06	.04	.07	.35	.56
Recommendation	-.09	-.04	.07	.36	.55
Injunctive belief	-.11*	-.06	.10	.43	.51
Control belief	-.11*	-.07	.09	.61	.44
High workload	.10*	.09	.05	2.68	.10
Sample 2012	.02	.03	.22	.02	.90
Sample 2013	.	.	.	.	.
*High intention vs. no clear decision (N = 319)*					
Instrumental attitude	.20**	.29	.11	7.28	.01
Experiential attitude	.14*	.09	.10	.87	.35
Injunctive norm	.10	-.00	.08	.00	.99
Descriptive norm	.11	.09	.09	1.12	.29
Capacity	.08	-.03	.07	.18	.67
Autonomy	.26**	.24	.11	5.19	.02
Self-protection	.04	-.03	.09	.13	.72
Patient protection	.02	-.06	.08	.55	.46
Recommendation	.07	-.00	.08	.00	.96
Injunctive belief	.02	-.04	.12	.08	.77
Control belief	.03	-.05	.11	.20	.65
High workload	-.03	.01	.06	.01	.91
Sample 2012	.02	-.08	.25	.11	.74
Sample 2013	.	.	.	.	.
Pseudo R^2^		.20			
Classification accuracy (%)		55.2			

*p < .05, two-tailed; **p < .01, two-tailed.

### Additional analyses

**Figure 1:**
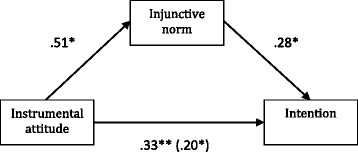
Regression coefficients for the relationship between instrumental attitude and intention to get vaccinated (no/ unsure) as mediated by injunctive norm. The path between instrumental attitude and injunctive norm is an OLS regression coefficient, while the other paths are logistic regression coefficients. The logistic regression coefficient between instrumental attitude and intention, controlling for injunctive norm, is in parentheses. *p < .05; ** p < .01.

In an exploratory manner we excluded the most influential variables autonomy and injunctive norm from the multinominal analysis and found that negative instrumental attitude became a significant predictor of no intention to get vaccinated as opposed to an unclear decision when injunctive norm was excluded. Therefore, we conducted a binary logistic regression using a bootstrapping technique [20] to analyse whether injunctive norm mediated the relationship between instrumental attitude and intention (no intention/ unsure; N = 420). The bias corrected and accelerated (BCa) confidence intervals were set at 0.95 with 5000 resamples. In the mediation analysis, instrumental attitude was the independent variable, intention (no/ unsure) was the dependent variable, and injunctive norm was the mediator. Results revealed a significant mediation effect of injunctive norm on the relationship between instrumental attitude and intention (b = .14, BCa 95% CI [.074; .225]; see Figure 1).

## Discussion

This cross-sectional study aimed at identifying the social cognitive variables that predict influenza vaccination intentions of German medical students and their reasons for refusal and acceptance of vaccination. We identified only few studies that focused exclusively on medical students [21-25]. Two of these studies report insufficient knowledge [21,22], while the other three studies focus on medical student’s reasons for acceptance and refusal of influenza vaccination [23-25]. In these cross-sectional studies, reported vaccination coverage rates range from 4.7 to 58.1%. In accordance, this study showed that only a small proportion of the medical students were motivated to get vaccinated against influenza in both samples (22% and 23% respectively) and that an even smaller proportion (12.9%) had been vaccinated in 2012. This is also consistent with the vaccination coverage rates of other HCP groups reported in European healthcare settings, including some of the studies about medical students [21,22,25-28]. However, some studies that included students had reported considerably higher vaccination rates [8,16,23]. Some studies had additionally found that clinical medical students are more likely to be vaccinated than pre-clinical students [23,24], while another study had found no difference between these two groups of students [22].

Reasons for accepting the vaccine found in previous studies were self-protection, patient protection, and that the vaccine was offered for free [23-25]. One study additionally found professional ethics, setting an example for patients, vaccine safety and the recommendation for HCP to get vaccinated as facilitating factors [24]. In the current study, the most common reasons reported for getting vaccinated against influenza were self-protection, patient protection and the protection of family and friends. Reasons for refusal of the vaccine reported in previous studies were inconvenience, forgetfulness, concerns about side-effects, perceiving vaccination as being unnecessary, and the cost of the vaccine. Students further indicated a low risk-perception, laziness and lack of knowledge [21-25]. Reported reasons for not getting vaccinated in the current study were mostly associated with a low risk-perception, fear of side-effects, and the disbelief in the effectiveness of influenza vaccination. To a lesser extent, organizational barriers were revealed to be a possible inhibiting factor, mirroring factors associated with refusal of influenza vaccination in other studies [8,23,28]. As was mentioned before, easy access to the vaccine is not always offered to medical students [7], even when they have regular patient contact.

Results further suggested that participants who did not expect important others to want them to get vaccinated were more likely to have no intention to get vaccinated as opposed to being unsure about their future vaccination intentions. Injunctive norm additionally mediated the relationship between instrumental attitude and intention. This is surprising since in the RAA, perceived norms and attitude predict intention independently [17]. Our findings suggest that medical students that have a negative instrumental attitude towards influenza vaccination might be even more susceptible to negative injunctive norms that they might encounter when entering a clinic, and that these two determinants predict their intention to get vaccinated. One possible explanation for this is that medical students are much more susceptible to injunctive norms in general because of their lower status in the healthcare hierarchy, as opposed to other HCP groups. This stresses the importance of intervening early in medical students’ education so that they form the right instrumental attitudes towards influenza vaccination before entering clinics. Moreover, higher feelings of autonomy in the decision whether to get vaccinated increased the probability of having no intention to get vaccinated as opposed to being unsure. A high intention to get vaccinated was most likely for participants who had a positive instrumental attitude and who also had high feelings of autonomy. Scores on autonomy were generally very high, suggesting that medical students feel completely free to choose whether to get vaccinated against influenza. High feelings of autonomy do not seem to be a barrier, as long as they are paired with positive instrumental attitudes.

The mentioned factors are significant but relatively weak predictors of the intention to get vaccinated against influenza. This indicates that there might be additional factors involved in the motivation of students to get vaccinated. The factors included in the multinominal logistic regression did not capture the organizational issues suggested in other studies, such as the inconvenience of getting vaccinated, not being offered vaccination, or getting vaccination for free [8,23]. However, previous studies have shown that these factors are perceived barriers to vaccination, and hospitals should therefore increase the accessibility of free vaccines to medical students and include them more actively in vaccination programs. This could also be an explanation for why the percentage of students who are vaccinated is smaller than the percentage of students who intend to get vaccinated against influenza. This intention-behavior gap has been identified across a broad range of health behaviors, including influenza vaccination [18,29].

This is one of the few studies to investigate the factors preceding the intention to get vaccinated among medical students. However, this study has some limitations worth mentioning. Firstly, the survey included only 18 items to reduce the length and to increase the response rate. Including more items that capture factors identified in other studies could have improved the predictive power of our model. Secondly, due to an unforeseen change in the set-up of the study, the second sample was missing questions on demographics, facilitating and inhibiting reasons, as well as the vaccination status of the participants. Therefore, we were only able to compare the intention to get vaccinated and its possible predictors across the two samples. We cannot say anything about differences in the number of people who were vaccinated. However, intention did not differ between the two years, making it likely that we would not have found considerable differences in vaccination rates between the two samples.

## Conclusions

In conclusion, this study showed that pre-clinical medical students have comparable knowledge gaps and negative attitudes with regard to influenza vaccination that have been reported among HCP already working in hospital settings. Education about influenza and vaccination should therefore be addressed early during medical training, and the importance of influenza vaccination should be taught so as to develop more favourable attitudes towards vaccination.
